# Predictors of treatment failure, time to switch and reasons for switching to second line antiretroviral therapy in HIV infected children receiving first line anti-retroviral therapy at a Tertiary Care Hospital in Ethiopia

**DOI:** 10.1186/s12887-019-1402-1

**Published:** 2019-01-29

**Authors:** Gelila Solomon Haile, Alemseged Beyene Berha

**Affiliations:** Department of Pharmacology and Clinical Pharmacy, School of Pharmacy , College of Health Sciences, Addis Ababa University, Addis Ababa, Ethiopia

**Keywords:** HIV-infected children, cART, HIV/AIDS, Treatment failure, Ethiopia

## Abstract

**Background:**

Treatment failure and delay in switching to second line regimen are major concerns in the treatment of HIV infected children in a resource limited setting. The aim of this study was to determine the prevalence and predictors of first line antiretroviral therapy (ART) regimen failure, reasons and time taken to switch to second line antiretroviral (ARV) medications after treatment failure among HIV-infected children.

**Methods:**

A retrospective cohort study was conducted February 2003 to May 2018 in HIV-clinic at Tikur Anbessa Specialized Hospital (TASH), Ethiopia. All HIV infected children ≤15 years of age and who were taking first line ART for at least 6 months were included. Data abstraction format was used to collect the data from patients’ chart and registry. Binary and multivariable logistic regression statistics were used.

**Results:**

Out of 318 enrolled HIV-infected children, the prevalence of treatment failure was found to be 22.6% (72/318), among these 37 (51.4%) had only immunologic failure, 6 (8.3%) had only virologic failure and 24 (33.3%) had both clinical and immunological failure. The mean time taken to modify combination antiretroviral therapy (cART) regimen was 12.67 (4.96) weeks after treatment failure was confirmed. WHO Stage 3 and 4 [Adjusted Odds Ratio (AOR), 3.64, 95% CI 1.76–7.56], not having both parents as primary caretakers [AOR, 2.72 95% CI, 1.05–7.06], negative serology of care takers [AOR, 2.69 95% CI, 1.03–7.03], and cART initiation at 11 month or younger were predicting factors of treatment failure. Of the 141 (47.9%) children who had regimen switching or substitution, treatment failure (44.4%) and replacement of stavudine (d4T) (30.8%) were major reasons. Only 6.6% patients had received PMTCT service.

**Conclusion:**

One fifth of the patients had experienced treatment failure. Advanced WHO stage at baseline, not being taken care of by mother and father, negative sero-status caretakers, and younger age at initiation of cART were the predictors of treatment failure. PMTCT service uptake was very low. There was a significant time gap between detection of treatment failure and initiation of second line cART. Half of the patients encountered regimen switching or substitution of cART due to treatment failure and replacement of stavudine (d4T).

## Background

At the end of 2016 there were approximately 36.7 million people worldwide living with HIV/AIDS, of these, 2.1 million were children (< 15 years old) and 70% (1.5 million) of children reside in Sub-Saharan Africa [1]. In Ethiopia there is a significant pediatric HIV-1 burden with approximately 65,100 infected children, with an estimated 3200 AIDS-related child deaths occurring annually [2].

The introduction of combination antiretroviral therapy (cART) has significantly decreased HIV associated morbidity and mortality. In Ethiopia the increased coverage of free cART program has enrolled hundreds of thousands of patients, with an overall cART coverage reaching 73% [3]. Improved factors like medication coverage, baseline CD4 count, and medication adherence over time have been linked to successful virological suppression [4, 5]. With all these documented progresses, treatment of HIV faces many challenges, out of these; treatment failure is a major concern. Basically HIV treatment failure occurs when a cART regimen is unable to control the HIV infection or unable to achieve the goals of therapy. Factors that can contribute to HIV treatment failure include drug resistance, drug toxicity, or poor adherence to antiretroviral therapy (ART). Failure could be detected either clinically, immunologically, or virologically [6]. It’s a threat according to the WHO, as it urges the global community for action against it [7]. A global study on the prevalence of treatment failure showed that low and middle income countries in Latin and sub-Saharan Africa regions were the highest to experience treatment failure [8].

In the current condition in Ethiopia where medication is fully funded by the government due to unaffordability to patients, treatment failure and frequent substitution of medications are a major setback to the economy [9]. According to *Médecins sans Frontières*, 2016 and a study conducted in the US in 2014, the cost of treating a patient with a second line ART drug increases by 24% as compared with the first line treatment [10, 11]. The higher the viral load the higher probability of passing HIV to another person, hence increasing government and personal health care cost [12]. Additionally the majority of patients have fewer adverse effects from initial medications they are started on compared to subsequent ones [13]. At individual patient level, compromised viral load suppression in treatment failure is a major risk factor for drug-resistance mutations, low CD4 T lymphocyte(CD4) cell numbers, and decreased clinical benefit of antiretroviral(ARV) medication [14] Uncontrolled viral load has been linked to decreased height and weight in children on cART, halting their expected developmental milestone for age [15]. Failed cART regimen limits treatment options, limits success of therapy and puts the patient at increased risk for drug toxicity from second-line regiments which have greater pill burden, and are more demanding for the child to adhere to compared with first-line regimens [16]. However if treatment failure has developed, timely switch to second-line regimes is very important. A delay in switch increases mortality and risk of developing opportunistic infections. By compromising the virological activity of standard second-line regimens, the chance of failing on treatment again is also high when there is delay to switch [17–20].

According to previous studies, treatment failure in HIV infected children is associated with different factors like infant ARV prophylaxis unavailability, TB infection at the time of diagnosis, substitution of initial cART regimen, duration of follow up of more than 60 month and poor adherence, WHO clinical stage 3 or 4, age group of 6 to 9 years, baseline CD4 count less than 50 cells/mm3, male gender, motherless children and stavudine containing regimen [21–24].

This is the first research output about treatment failure and its predictors in HIV-infected children who were on failing first-line ART regimen during the era of HIV ‘test and treat’ strategy, expansion of viral load testing and switch of first line regimens from stavudine(d4T) to zidovudine(AZT) and tenofovir(TDF) based regimens in Tikur Anbessa Specialized Hospital(TASH), Addis Ababa, Ethiopia. Most of the similar studies conducted in Ethiopia used CD4 count and clinical stages as the major tools to assess treatment failure, which are known to have poor sensitivity and specificity to detect treatment failure. The availability of regular viral load testing for detection of treatment failure and implementation of the revised Ethiopian national guideline for changing of initial CARTcART regimens is claimed to improve health care service in TASH. The aim of this study was to determine the prevalence and predictors of first line antiretroviral therapy (ART) regimen failure, reasons for switching second line antiretroviral (ARV) drugs and time taken to switch to second line ARV drugs after treatment failure among HIV-infected children at Tikur Anbessa Specialized Hospital (TASH), Ethiopia.

## Methods

### Study area

Tikur Anbessa Specialized Hospital (TASH) is a tertiary care teaching hospital in Ethiopia, with over 700 beds. The data was collected in the HIV- pediatric clinic of department of pediatrics and child health. Based on the 2018 health management information system (HMIS) data, currently 450 HIV-infected children ranges from 0 to 19 years on follow up.

#### Study design and period

A retrospective cohort study design was used to collect the data from HIV-infected pediatric patients’ medical chart and HMIS registry. This study included all children 15 years or younger who were on ART regimen between February 2003 to May 2018.

### Data collection procedure

Data was collected from 318 patients in TASH HIV clinic. Children seen at this center were closely followed based on the Ethiopian national guideline which recommends that children should be evaluated 2 weeks after initiation of cART, every month for the next 2 months, and every 3 months afterward. To be included in this study, all HIV infected children ≤15 years of age and children who had a minimum of two follow-up visits with at least one visit six months post initiation of ART were included in the sample. Children who took ART only for prevention of mother to child transmission (PMTCT) were excluded. According to this study, HIV related poor clinical outcomes comprised treatment failure and death. Adherence to cART was assessed at every follow-up visit and calculated by using brief survey of missed doses in the last 3 days, 7 days or two weeks can be used out of expected monthly number of doses. Adherence rate defined as “good” “fair” or “poor” if the self-reported number of doses was greater than or equal to 95% (skipping < 2 doses out of 30 doses), between 85 and 94% (skipping 3–5 doses out of 30 doses) or less than 95%(skipping > 6 doses out of 30 doses), respectively from expected monthly number of doses.

### Data analysis

HIV-infected children data was abstracted from patient medical chart using a pretested structured questionnaire. Age at cART initiation, adherence, base line CD4 count, baseline WHO staging, initial cART medication, parental status, initial cART regimen and dosing preparation, serology of care taker, and types of primary care taker were collected by reviewed medical charts. The questionnaires were filled by the nurses at the pediatric HIV clinic who were given training by the researcher on the purpose, procedure and data collection technique of the study. In addition to the practical training, adequate supervision and follow-up was done by the r researcher to assure quality of the data collected. Data was entered, analyzed using statistical package for social sciences (SPSS) version 21.0 for analysis. Descriptive statistics were performed to present the frequency, percentage, mean, range and standard deviation (SD).To identify factors associated with treatment failure, first univaraite binary logistic regression was done and independent variables that showed a *p*-value of less than 0.25 were taken to the multiple logistic regression analysis. Then association between the outcome and the independent variables was taken as significant at *P* < 0.05 in the multiple logistic regression analysis.

## Results

### Socio-demographic characteristics

Out of 450 HIV-infected children who were treated at the pediatric HIV clinic of TASH from February 2003–May 2018, 391 children who fulfilled the inclusion criteria were included in the study. Seventy three (18.7%) children were excluded from the study due to missing data (47, 12.0%) and died (26, 6.6%) during the follow up period. Out of the children being studied (318), 72 (22.6%) of them encountered a treatment failure. From the total, 181 (56.9%) were male and 242 (77%) were between the age group 10 to15 years at the time of the study. The mean age at time of study was 12.26 (± 2.71). Majority, 217(68.2%), of the children lived with at least one of their biological parents. More than half (59.4%) of the care givers were positive HIV serologic status (Table 1).

**Table 1:** Socio-demographic characteristics of HIV infected children in HIV clinic at Tikur Anbessa Specialized Hospital, Addis Ababa, Ethiopia from April 1 to May 15, 2018 (*n* = 318)

	Patient characteristics	N (%)
Age years, mean SD 12.32(± 2.65)		
0–5 years		8(2.5)
6–9 years		65(20.4)
10–15 years		245(77)
Sex		
Male		181 (56.9)
Female		137 (43.1)
Parental Status		
Both Alive		128(40.3)
Either dead		98(30.8)
Both dead		73(23)
Unknown		19(6)
Primary care taker		
Both Parents		99(31.1)
Mother		70(22)
Father		48(15.1)
Relatives		46(14.5)
Guardian/Neighbors		1(0.3)
Orphanage		54(17)
Serology of care taker		
Positive		189(59.4)
Negative		51(16)
Unknown		78(24.5)

### Baseline characteristics

Twenty one (6.6%) patients had received PMTCT service. Almost half of the patients (48.7%) were WHO stage 3 at baseline diagnosis. The mean CD4 percentage at medication initiation was 13.7% (± 3.3%) for those who were less than 5 years old. Half of the children, 155 (48.7%) were found to be in severe immunosuppression at the time of cART initiation. None of the children had their baseline viral load tested. The mean age at which medication was started was 2.63 (± 1.23) years. One third (32.3%) of the patients had started their cART medication with AZT-3TC-NVP regimen (Table 2).

**Table 2:** Baseline characteristics of HIV infected children in HIV clinic at Tikur Anbessa Specialized Hospital, Addis Ababa, Ethiopia from April 1 to May 15, 2018 (*n* = 318).Baseline characteristics of

Variables	N (%)
Age years (at the time of cART initiation)	
≤ 11 months	91(28.6)
12 to 34 months	63(19.8)
35–59 months	52(16.4)
≥ 60 months	112(35.2)
Initial HAART Regimen	
D4T-3TC-NVP	59(18.5)
D4T-3TC-EFV	17(5.3)
AZT-3TC-NVP	103(32.3)
AZT-3TC-EFV	53(16.6)
TDF-3TC-EFV	26(8.1)
AZT-3TC-LPV/r	10(3.1)
ABC-3TC-LPV/r	6(1.5)
ABC-3TC-EFV	23(7.2)
D4T-3TC-LPV/r	21(6.6)
Initial cART dosing preparations	
One loose	94(29.6)
Three loose	81(25.5)
Fixed	143(45)
PMTCT Service	
Present	21(6.6)
None	237(74.5)
Unknown	60(18.8)
Infant ART Prophylaxis	
Yes	37(11.6)
No	235(73.8)
Unknown	56(17.6)
Baseline Immunosuppression level	
Not Significant Immunosuppression (≥ 500 or > 25%)	3(0.94)
Mild Immunosuppression (350–499 or 20–24%)	23(7.2)
Advanced Immunosuppression (200–349 or 15–19%)	137(43.08)
Severe Immunosuppression (< 200 or < 15%)	155(48.74)
Base line WHO staging	
Stage 1	26(8.17)
Stage 2	109(34.3)
Stage 3	155(48.7)
Stage 4	28(8.8)

### Current follow-up data

The mean follow up period after ART initiation in the current study was 100.7(±40.93) months, with the range of 14 to 181 months.. Adherence to ART for the last 6 months of follow-up was assessed and the finding of this study showed that majority (84.5%) of participants had “good” adherence status. Almost half (50.9%) of patients were substituted or switched from first line to the other ARV medications or cART regimen due to treatment failure 72(44.4%) and stavudine substitution (d4T) 50(30.8%). Out of 72(22.6%) treatment failed patients with a mean duration of 110(± 38.89) months, 37 (51.4%) had only immunologic failure, 6 (8.3%) had only virologic failure and 24 (33.3%) had both clinical and immunological failure. A mean of weeks to initiate second line ARV regimens was 12.67 (± 4.96) after treatment failure developed. Twenty six (36.1%) patients were switched to second line cART regimen within 30 days. Out of 318 patients, 289 (90.8%) patients had a WHO T1 stage diagnosis during the study period at enrollment and follow up of which 241 (75.8%) patients had mild to insignificant immunosuppression based on their current CD4 count. There was successful viral load suppression to undetectable level for 268(84.2%) pediatric patients. Out of the children being fulfilled the inclusion criteria, 26 (6.6%) had died during follow up (See in Table 3).

**Table 3:** Follow-up data of HIV infected children in HIV clinics at Tikur Anbessa Specialized Hospital, Addis Ababa, Ethiopia from April 1 to May 15, 2018 (*n* = 318)

Variables		N (%)
Status Category		
Alive on ART		318 (92.4)
Dead		26 (7.6)
Duration of follow up(month)		
≤ 36		13 (4.1)
37–59		31 (9.7)
≥ 60		274 (86.2)
Substitution of first line		
Yes		162 (50.9)
Treatment failure		72 (44.4)
New drug available		50 (30.8)
Toxicity		23 (14.1)
Drug stock out		6 (3.7)
Change to fixed dose		6 (3.7)
New TB diagnosis		2 (1.23)
Others		3 (1.85)
No		156 (49.05)
Time taken to initiate second line medication		
< 4 weeks		26 (36.1)
≥ 5 weeks		46 (63.8)
Adherence status of past 6 month		
Good		269 (84.5)
Fair		37 (11.6)
Poor		12 (3.7)
WHO T stage		
T1		289 (90.8)
T2		7 (2.2)
T3		1 (0.31)
T4		21 (6.6)
Current CD4 count		
Not-significant immunosuppression		241(75.8)
Mild-immunosuppression		49 (15.4)
Advanced-immunosuppression		17 (5.3)
Severe-immunosuppression		11 (3.5)
Viral load test		
Undetectable		268 (84.2)
≥ 150		24 (7.5)
Missing data		26 (8.1)
Developed treatment failure to first line		
Yes		72 (22.6)
Clinical failure only		2 2.7
Immunological failure only		37 (51.3)
Virologic failure only		6 (8.3)
Clinical and immunological		24 (33.3)
Virologic and immunologic		3 (4.17)
No		246 (77.4)

### Factors associated with treatment failure

Binary logistic regression was used to identify independent determinants for treatment failure with a *p*-value of less than 0.25 such as not having both parents as primary caretaker, negative and unknown serology of the caretakers, non-disclosed to the child, baseline WHO stage 3 and 4 and initiation of cART at 11 months or less were selected as potential predictors for further analyses. In binary logistic regression, there was no association between treatment failure with child sex, initial cART regimen, adherence status, disclosure to the child and baseline CD4 T cell count or percentage. Multiple logistic regression analysis was performed to assess independent predictors of treatment failure. Respected to this, it was found that patients not having both parents as a primary care taker, negative serology of the caretakers and baseline WHO stage 3 and 4 were significantly associated with treatment failure. Age at initiation of cART between 12 and 34 months and more than 60 months was significantly associated with less likely of treatment failure (See in Table 4).

**Table 4:** Predicting of first line HAART failure in HIV infected children with HIV/AIDS treated in HIV clinics at Tikur Anbessa Hospital, Addis Ababa, Ethiopia from April 1 to May 15, 2018.(*n* = 318)

Independent variable	Treatment Failure (%)	Treatment Success (%)	Odds Ratio (95% CI)	Adjusted Odds Ratio (95%CI)^a^
Primary Care Taker				
Both parents	8(8.08)	91(91.92)	1.00	
Other Care takers	64(29.22)	155(70.77)	3.72 (1.61–8.61)	2.72 (1.05–7.06)*
Serology of Care Taker				
Positive	32(16.90)	157(83.06)	1.00	
Negative	15(29.41)	36(70.50)	2.27 (1.03–4.99)	2.69 (1.03–7.03)*
Unknown	25(32.05)	53(67.95)	2.12 (1.05–4.27)	2.26 (0.93–5.49)
Disclosure to the Child				
Disclosed	57(20.2)	225(79.7)	1.00	
Not disclosed	15(41.6)	21(58.40)	2.48 (1.09–5.65)	1.41 (0.55–3.65)
Base Line WHO Stage				
Stage 1 and 2	20(10.9)	162(89.01)	1.00	
Stage 3 and 4	52(38.2)	84(61.70)	5.33 (2.73–10.40)	3.64 (1.76–7.56)*
Age at initiation of HAART				
< =11 month	37(40.6)	54(59.30)	1.00	
12–34 months	14(22.22)	49(77.78)	0.39 (0.17–0.88)	0.40 (0.16–0.99)*
35–59 months	14(26.92)	38(73.08)	0.49 (0.21–1.14)	0.55 (0.21–1.43)
> = 60 months	7(6.25)	105(93.75)	0.07 (0.02–0.21)	0.07 (0.02–0.24)*

*- Variables that showed a *p*-value of less than 0.05 on the multiple logistic regression analysis

^a^Adjusted odds ratio – adjusted for primary care taker, serology of care taker, disclosure to the child, base line WHO stage and age at the initiation of HAART

## Discussion

This study found that 72(22.6%) treatment failure from a total of 318 enrolled HIV infected children who were on first line cART regimen. Out of this, immunologic failure was the most common followed by both clinical and immunological failure as well only small proportion confirmed with virologic failure.

In the present study, only 6 (8.3%) patients had confirmed virologic failure,. Contrary to this study, results reported by Zoufaly et al. in 2013 showed that in 53% of the patients had experienced virologic failure [25]. In addition, in a cross sectional study conducted in Central Africa Republic 58% of the children were found to have encountered virologic failure [26]. The low level of occurrence of virologic failure in this study might be due to two reasons. The first thing about the availability viral load testing is limited in Ethiopia. The second is that the healthcare providers who strictly wait the viral load to reach a minimum of 1000copies/ml to detect virologic failure. More importantly the WHO recognizes that this threshold has not been proven to be optimal for detecting treatment failure [27].Various studies have proven that this threshold significantly misclassifies patients and undermines the large subset of patients who require clinical intervention [28, 29]. In this particular study 24(7.5%) of the patients had detectable viral load (≥ 150 copies /ml) but have not been given attention regarding this.

In the current study, children at baseline with WHO stage 3 and 4 had 3.64 times chance of failing on first line regimen when compared with children at stage 1 and 2. The finding of this study similar with study done in Ethiopia Oromiya region identified that patients at WHO stage 3 and 4 had more than twice the chance of failing treatment than stage 1 and 2 [22]. Another study conducted in Uganda and Mozambique reported that patients with advanced WHO stage at baseline were 1.57 time more likely to experience treatment failure [30]. In addition it was one of the main predicting factors of treatment failure in a study conducted in Ethiopia Jimma town, with patients having 5 times more chance of failing treatment [31].

The current study found that children not taken care of by both parents were at increased risk of treatment failure. Similarly a study on the importance of caregivers in the outcome of pediatric HIV management in Kenya found that treatment failure was associated with not having both parents as caregivers [32]. This might be due to the fact that children taken care of by both parents have a much better health and developmental outcomes. According to the National Health Interview Survey done in the U.S, children not taken care of by both parents reported poor health outcomes more frequently than children that lived with both of their parents [33]. This children are also at increased risk of psychological distress [34]. Another study stated that children living with both of their biological parents on a regular basis had better health, emotional, and physical well-being [35].

In the current study, negative sero-status of care takers was a predicting factor of treatment failure. In contrast to this study, a study conducted in South Africa and Malawi in 2013 showed that positive serology of the care taker related with poor health outcome of the child being taken care of [36]. The results from this study might be due to the inadequate understanding of the caretakers who have HIV negative. A study conducted in west and sub-Saharan Africa showed that individuals who were HIV negative had lower understanding and knowledge of the disease when compared to people who lived with the virus [37]. Another study done by Woodward et al. in 2000*,* showed that HIV positive individuals had better information regarding nutrition, alarming sign and symptoms, importance of medication adherence and emotional and psychological aspect of the disease [38]. Hence not having this understanding of the disease by the HIV negative care takers might have led to poor management of disease related situation in the children. Further research on socio-demographic and awareness level on caretakers should be done as it is a major predicting factor.

Younger age at cART initiation was found to be a predicting factor for treatment failure in this study. Patients who had started at 11 month or younger had an increased chance of failure. Children started on medication in the age group 12–34 months and ≥ 60 months were less likely to fail treatment. Similar studies had reported different results regarding the association between age and treatment failure. A study conducted in Tanzania and four referral hospitals in Ethiopia had similar a finding, the younger the child started cART the higher the chance of failing treatment [23]. But a study done by Puthanakit et al. in 2009 reported that younger age at the time of cART initiation was a protecting factor of treatment failure [39]. According to Kuhn et al. in 2018, children that were initiated on cART between 2 and 4 month had a decreased the chance of failure, on the other hand initiation at ≥ 5 month increased the chance of failure [40]. The reason for the variation of these results could not be identified.

In practice, switch delayed if at all [41], with the present study, it took a mean of 12.67 (± 4.96) weeks to modify cART regimen after treatment failure was detected, with a 36.1% patient’s regimen being changed within 30 days. Whereas, significantly shorter time was taken to switch to second line cART regimens in this study compared with other studies [23, 30, 42–44]. This could be due to the availability of viral load testing in the study setting during the study period, unlike the other studies. This is justified by studies showed that HIV clinics that conduct routine viral load testing take shorter amount of time to switch to second line when compared to clinics that don’t [45, 46]. In contrast, study done in Ethiopia Oromiya region in 2017 reported that a smaller time gap was taken to modify cART, with 75% of the patients being started on second line within 30 days of treatment failure detection [22]. In this study, results showed that there is a notable delay in time taken to modify regimen after failure was confirmed according to the WHO guidelines [20]. This could be due to the use of only immunological and clinical criteria as detection of failure until recent years, which often leads to a delayed initiation of second line medication [23]. The delay to switch to second line regimen has to be addressed by the clinic due to its damaging consequences.

In the current study, multiple reasons have led to the substitution or changing of cART regimens for 162(50.9%) patients. Among these, treatment failure had the largest share with 72(44.4%) patients, followed by the substitution of all stavudine (d4T) to zidovudine (AZT) or tenofovir disoproxil fumarate (TDF) based regimens in 50 (30.8%) patients since the implementation of the revised Ethiopia National Guidelines for Comprehensive HIV Prevention, Care and Treatment, 2014 [47]. In addition, substitution due to toxicity was from all d4T and AZT based regimens in 23 (14.1%) patients. Likewise in a retrospective study conducted on children that received cART for at least six months in a tertiary hospital in Malaysia in 2018 reported the major reasons for substituting medications were treatment failure and drug toxicity in 39 (54.9%) and 14(19.7%), respectively [48]. Also a study in Swaziland in 2012 had similar findings to this study in that d4T regimen change was major reason due to its toxicity or guideline change in 105(77%) patients [49].

Though statistically insignificant, only 21(6.6%) patients had received PMTCT service. Similarly in a retrospective cohort study conducted in Ethiopia Oromiya region in 2017, 16 (5.9%) patients had received PMTCT service [22]. PMTCT service in this study was higher compared to results from other previous similar setting studies [21, 23] .This might be due to the scale-up of Option B+, an implementation of the “test and treat”strategy in HIV+ pregnant women, since 2013 by the Ethiopia Ministry of Health [50].

## Conclusion

One fifth of the patients had experienced treatment failure. Advanced WHO stage at baseline, not being taken care of by mother and father, negative sero-status caretakers, and younger age at initiation of cART were the predictors of treatment failure. PMTCT service uptake was very low. There was a significant time gap between detection of treatment failure and initiation of second line cART regimens. Half of the patients encountered regimen switching or substitution of cART due to treatment failure and replacement of stavudine(d4T).

## Abbreviations

3TC: Lamivudine
ABC: Abacavir
AIDS: Acquired immunodeficiency syndrome
ART: Anti-retroviral therapy
ATZ: Atazanavir
AZT: Zidovudine
CD4 +: T-lymphocyte bearing CD4 receptor
CDC: Centers for Disease Control and Prevention
d4T: Stavudine
DNA: Deoxyribonucleic acid
EFV: Efavirnez
cART: Highly active anti-retroviral treatment
HIV: Human immunodeficiency virus
LPV/r: Lopenavir/ritonavir
NVP: Nevirapine
PMTCT: Prevention of Mother to Child Transmission (of HIV)
TDF: Tenofovir
WHO: World Health Organization
